# Estimating effects of intervention measures on COVID-19 outbreak in Wuhan taking account of improving diagnostic capabilities using a modelling approach

**DOI:** 10.1186/s12879-021-06115-6

**Published:** 2021-05-05

**Authors:** Jingbo Liang, Hsiang-Yu Yuan, Lindsey Wu, Dirk Udo Pfeiffer

**Affiliations:** 1 Department of Biomedical Sciences, Jockey Club College of Veterinary Medicine and Life Sciences, City University of Hong Kong, Hong Kong, China; 2 Department of Infection Biology, Faculty of Infectious and Tropical Diseases, London School of Hygiene & Tropical Medicine, London, UK; 3 Centre for Applied One Health Research and Policy Advice, City University of Hong Kong, Hong Kong, China

## Abstract

**Background:**

Although by late February 2020 the COVID-19 epidemic was effectively controlled in Wuhan, China, estimating the effects of interventions, such as transportation restrictions and quarantine measures, on the early COVID-19 transmission dynamics in Wuhan is critical for guiding future virus containment strategies. Since the exact number of infected cases is unknown, the number of documented cases was used by many disease transmission models to infer epidemiological parameters. This means that it was possible to produce biased estimates of epidemiological parameters and hence of the effects of intervention measures, because the percentage of all cases that were documented changed during the first 2 months of the epidemic, as a consequence of a gradually improving diagnostic capability.

**Methods:**

To overcome these limitations, we constructed a stochastic susceptible-exposed-infected-quarantined-recovered (SEIQR) model, accounting for intervention measures and temporal changes in the proportion of new documented infections out of total new infections, to characterize the transmission dynamics of COVID-19 in Wuhan across different stages of the outbreak. Pre-symptomatic transmission was taken into account in our model, and all epidemiological parameters were estimated using the Particle Markov-chain Monte Carlo (PMCMC) method.

**Results:**

Our model captured the local Wuhan epidemic pattern as two-peak transmission dynamics, with one peak on February 4 and the other on February 12, 2020. The impact of intervention measures determined the timing of the first peak, leading to an 86% drop in the R_e_ from 3.23 (95% CI, 2.22 to 4.20) to 0.45 (95% CI, 0.20 to 0.69). The improved diagnostic capability led to the second peak and a higher proportion of documented infections. Our estimated proportion of new documented infections out of the total new infections increased from 11% (95% CI 1–43%) to 28% (95% CI 4–62%) after January 26 when more detection kits were released. After the introduction of a new diagnostic criterion (case definition) on February 12, a higher proportion of daily infected cases were documented (49% (95% CI 7–79%)).

**Conclusions:**

Transportation restrictions and quarantine measures together in Wuhan were able to contain local epidemic growth.

**Supplementary Information:**

The online version contains supplementary material available at 10.1186/s12879-021-06115-6.

## Introduction

Coronavirus disease 2019 (COVID-19), an acute respiratory infection originally identified in the city of Wuhan in Hubei Province, China, has spread worldwide in 2020 [1, 2]. Estimating the effects of intervention measures is still one of the major scientific goals in order to identify prevention measures that are effective in different countries around the world [3]. The precise estimation of the effective reproduction number (R_e_), the expected number of new infections caused by an infectious individual, is critical for the identification of appropriate intervention measures to contain the outbreak [1, 4–8]. Although many recent studies have evaluated how intervention measures implemented in Wuhan reduced disease spread to regions outside Wuhan [6, 9–12], the investigation of the contribution of interventions within Wuhan, the epidemic source region itself, has received little attention [13, 14], possibly because an irregular pattern of transmission dynamics during early February hinders the model fitting processes, making the precise estimation of the parameters difficult.

To control the virus spread during the early outbreak stage, the Chinese government implemented strict travel restrictions on January 23, 2020 in Wuhan [15]. The first epidemic peak occurred 12 days after the restrictions were implemented. Soon afterwards, the number of new daily documented cases started to fluctuate for about 2 weeks around this peak value, followed by another peak with an extremely high number of cases, and it then reduced to very low levels (Figure S1). The transmission dynamics with such an irregular and unusual pattern can affect the estimation of the effects of intervention measures. The high number of documented cases after the introduction of interventions was generally hypothesized to be mainly caused by improved diagnostic capability [16], leading to more detected cases rather than caused by the intrinsic growth of the epidemic. However, most studies have not considered the changes in diagnostic capability over time, which can affect the number of documented infections and, ultimately, the estimation of R_e_.

**Fig. 1:**
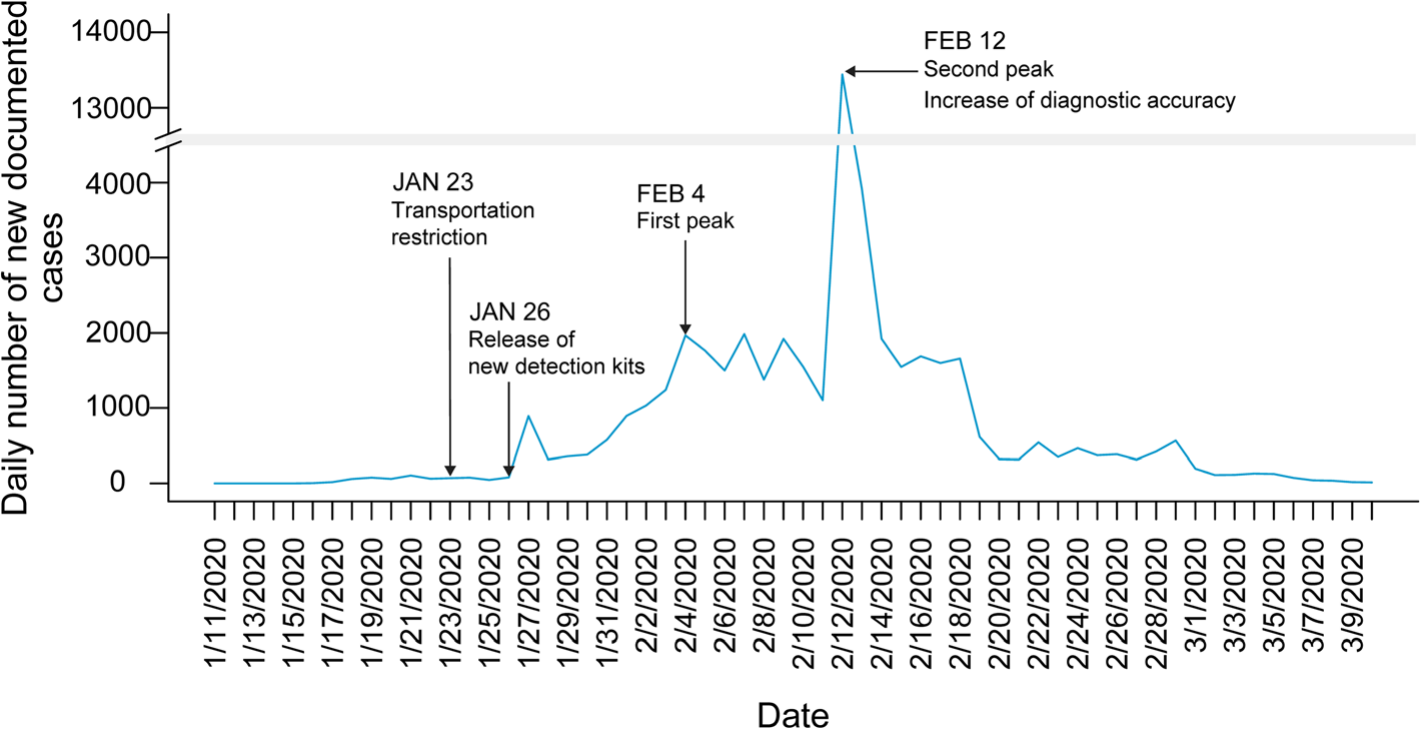
The daily number of new COVID-19 documented (reported) cases by date and the timeline of improved diagnostic capability and transportation restrictions implemented in Wuhan, China. Wuhan transportation restrictions were implemented on January 23 [15]; New commercial kits were approved by the State Food and Drug Administration (SFDA) on January 26 [18]; Updated diagnostic criteria, i.e. COVID-19 case confirmation should rely on both clinical diagnosis and laboratory diagnosis, was introduced on February 12 [16]. A break was made in the y-axis, and the narrow grey horizontal bar indicates where the break was set

Accounting for temporal changes in COVID-19 diagnostic capability is critical for characterizing transmissibility and understanding the pattern of the local Wuhan epidemic. Recent studies have shown that the total potential number of cases has been significantly underestimated, with more than 80% of all infections undocumented during the initial period following the identification of SARS-CoV-2 as the causative agent [17]. While the number of total new infections is driven by the epidemic growth, after the introduction of new commercial kits [18] and introduction of more sensitive diagnostic criteria [16] (Fig. 1), diagnostic capacity in Wuhan improved, resulting in a higher proportion of total new infections been documented. Therefore, it is important to consider the improvements in diagnostic capacity over time when using the documented data to construct transmission models for COVID-19 in Wuhan.

A particularly important challenge is to understand the proportion of transmission that occurs prior to the onset of illness. During the early outbreak, several studies have described the pre-symptomatic transmission of SARS-CoV-2, including a 20-year-old woman from Wuhan believed to have passed on the infection to five of her family members [19] and a Chinese individual believed to have infected her German business partner [20], both in the absence of symptoms. The existence of pre-symptomatic transmission indicates that COVID-19 infected individuals can be infectious during the incubation period. However, previous classical susceptible-exposed-infected-recovered (SEIR) models assume weak or no infectiousness during the incubation period [14, 21], potentially resulting in an underestimation of the infectiousness of COVID-19 cases.

In this study, in order to overcome the difficulties related to describing irregular fluctuations in the transmission dynamics and the limitation of the simple SEIR model for dealing with such data, a stochastic susceptible-exposed-infected-quarantined-recovered (SEIQR) model was developed to describe the Wuhan COVID-19 transmission pattern after the initial outbreak stage. This model extends the classic SEIR model by including pre-symptomatic transmission and quarantined status and allows the effects of transportation restrictions and quarantine measures on virus transmission patterns to be estimated while accounting for improvements in the diagnostic capacity over time. After considering varying diagnostic capabilities, we will show that this model can capture the transmission dynamics well and can estimate the reduction in R_e_ precisely.

## Methods

### Data collection

The daily number of new documented COVID-19 confirmed cases from January 11 to March 10 in Wuhan, Hubei province, China, by reported date, were collected from the Wuhan Municipal Health Commission [22] and the National Health Commission of the People’s Republic of China [23]. During this period, asymptomatic cases were not classified as confirmed cases in Wuhan [24, 25], and only confirmed cases were reported in the commission’s official daily reports.

### Description of the SEIQR epidemic model

**Fig. 2:**
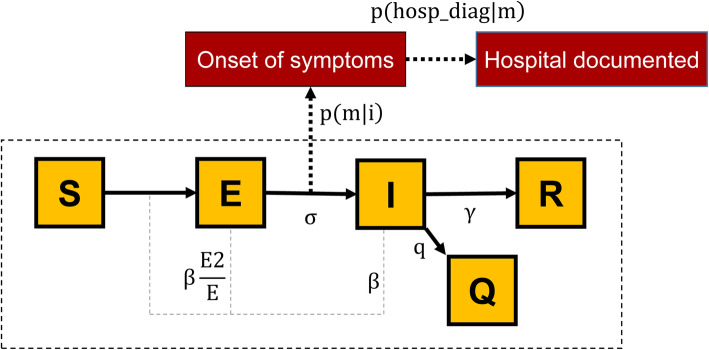
SEIQR model schema. The population is divided into five compartments: S (susceptible), E (exposed), I (infectious), Q (quarantined), and R (recovered). E2 is the number of exposed individuals after latent period who are pre-symptomatically infectious, β is the transmission rate, σ is the incubation rate, q is the quarantine rate, γ is the recovery rate. A fraction of newly symptomatic infections seek for medical care and are eventually documented by hospitals, where p(m| i) is the probability of an infection develops symptoms and seeks medical care, p(hosp _ diag| m)_t_ represents the probability that a symptomatic infectious outpatient is diagnosed as COVID-19 case by the hospital

An SEIQR model was developed to estimate the effect of intervention measures on COVID-19 transmission dynamics in the Wuhan population (Fig. 2). In our model, S, E, I, Q and R represent the number of individuals in susceptible, exposed, infectious (after incubation time), quarantined, and recovered statuses, with the total population size N = S + E + I + Q + R assumed to be 11 million (the permanent population in Wuhan [26]). Here, we defined susceptible individuals change to exposed individuals after they have had effective contact with the virus. Exposed individuals were further divided into two groups: E1, exposed individuals at the latent period who are not able to transmit the disease; E2, exposed individuals not at the latent period who are at a pre-symptomatic stage (referred to pre-symptomatically infectious individuals). The proportions of E1 and E2 out of total exposed individuals were determined using the proportion of the time span of latent period and pre-symptomatic transmission period within the incubation period. The SEIQR equations were derived as follows:

1 $$ {\displaystyle \begin{array}{c}{\mathrm{S}}_{\mathrm{t}}={\mathrm{S}}_{\mathrm{t}-1}-{\Delta}_{\mathrm{E},\mathrm{t}}\\ {}{\mathrm{E}}_{\mathrm{t}}={\mathrm{E}}_{\mathrm{t}-1}+{\Delta}_{\mathrm{E},\mathrm{t}}-{\Delta}_{\mathrm{I},\mathrm{t}}\\ {}\begin{array}{c}{\mathrm{I}}_{\mathrm{t}}={\mathrm{I}}_{\mathrm{t}-1}+{\Delta}_{\mathrm{I},\mathrm{t}}-{\Delta}_{\mathrm{R},\mathrm{t}}-{\Delta}_{\mathrm{Q},\mathrm{t}}\\ {}\begin{array}{c}{\mathrm{Q}}_{\mathrm{t}}={\mathrm{Q}}_{\mathrm{t}-1}+{\Delta}_{\mathrm{Q},\mathrm{t}}\\ {}{\mathrm{R}}_{\mathrm{t}}={\mathrm{R}}_{\mathrm{t}-1}+{\Delta}_{\mathrm{R},\mathrm{t}}\end{array}\end{array}\end{array}} $$

Δ_E, t_ is defined as the number of newly exposed individuals before symptom onset, during a time interval from t − 1 to t, Δ_I, t_ is the number of new infections after incubation time (including both symptomatically and asymptomatically infectious cases), Δ_Q, t_ is the number of newly quarantined cases, and Δ_R, t_ is the number of newly recovered individuals. We assumed Δ_E, t_, Δ_I, t_, Δ_Q, t_, and Δ_R, t_ follow Poisson distributions:

2 $$ {\displaystyle \begin{array}{c}{\Delta}_{\mathrm{E},\mathrm{t}}\sim \mathrm{Poisson}\left(\frac{\upbeta_{\mathrm{t}-1}\left[\mathrm{E}{2}_{\mathrm{t}-1}+{\mathrm{I}}_{\mathrm{t}-1}\right]{\mathrm{S}}_{\mathrm{t}-1}}{\mathrm{N}}\right)\\ {}{\Delta}_{\mathrm{I},\mathrm{t}}\sim \mathrm{Poisson}\left(\upsigma \times {\mathrm{E}}_{\mathrm{t}-1}\right)\\ {}\begin{array}{c}{\Delta}_{\mathrm{Q},\mathrm{t}}\sim \mathrm{Poisson}\left(\mathrm{q}\times {\mathrm{I}}_{\mathrm{t}-1}\right)\\ {}{\Delta}_{\mathrm{R},\mathrm{t}}\sim \mathrm{Poisson}\left(\upgamma \times {\mathrm{I}}_{\mathrm{t}-1}\right)\end{array}\end{array}} $$

where E2_t − 1_ is the number of pre-symptomatically infectious individuals (E2) at time t − 1, assumed determined as $\mathrm{E}{2}_{\mathrm{t}-1}=\left(\frac{\frac{1}{\upsigma}-\upeta}{\frac{1}{\upsigma}}\right){\mathrm{E}}_{\mathrm{t}-1}$, σ is the rate at which some exposed individuals become symptomatically infectious cases (1/σ is the incubation period), η is the latent period, q is the quarantine rate (1/q the time between symptom onset and quarantine start), γ is the recovery rate, expressed by γ = 1/(τ − 1/σ), and τ is the generation time. Here we assumed τ was fixed to be 10 days considering the period from being infected to recovered was generally longer than the observed serial interval (e.g. 7.5 days) [1] and the infectious period was estimated to be around 10 days by a virology study [27]. Using a constant value of τ can reduce the model uncertainty. β_t_ is the transmission rate on day t. In this model, β_t_ is assumed to be modulated by the Wuhan transportation restriction policy, represented as an exponential relationship with a lag effect:

3 $$ {\upbeta}_{\mathrm{t}+\mathrm{lag}1}={\mathrm{e}}^{\left(\upalpha \times {\mathrm{pol}}_{\mathrm{t}}+\log \left({\upbeta}_0\ \right)\right)} $$

where pol_t_ is an indicator variable for the daily transportation restriction policy, with pol_t_ = 0 if there is no transportation restriction at time t (i.e., before January 23) [15] and pol_t_ = 1 otherwise. α is the transportation restriction effect coefficient, β_0_ is the basic transmission rate without transportation restrictions, and lag1 indicates the lag time of the transportation restrictions effect on the virus transmission rate assumed to be 6 days [13]. Thus, β_t_ has a constant value throughout the period before the transportation restriction worked and change to a different constant value after then.

### Mapping SEIQR model to observed hospital document cases

Model estimates of new infections (Δ_I, t_, including both symptomatically and asymptomatically infectious cases) can not be compared with observed hospital documented cases directly. This is because documented data only captures COVID-19 cases who seek hospital care and are successfully diagnosed, which will only be a proportion of the total number of new infections in the population estimated in the model. To address this discordance, we introduced an observation model to link the SEIQR model simulated new infections to the observations. The daily number of hospital documented cases, (hosp _ document)_t + lag2_, was assumed to follow a normal distribution with the mean defined as the number of new infections Δ_I, t_ that were reported (documented) with a delay of lag2 (days). Here, lag2 was a parameter, which was set as 6 (days) [13]:

4 $$ {\left(\mathrm{hosp}\_\mathrm{document}\right)}_{\mathrm{t}+\mathrm{lag}2}\sim \mathrm{Normal}\left({\Delta}_{\mathrm{I},\mathrm{t}}\times \mathrm{p}\left(\mathrm{m}|\mathrm{i}\right)\times \mathrm{p}{\left(\mathrm{hosp}\_\operatorname{diag}|\mathrm{m}\right)}_{\mathrm{t}+\mathrm{lag}2},{\epsilon}^2\right) $$

where p(m| i), the probability of an infection develops symptoms and seeks medical care, was assumed to be fixed at 0.8 according to the high motivation of care-seeking behavior in Wuhan [28]. Hospital diagnostic rate, p(hosp _ diag| m)_t + lag2_, represents the probability that a symptomatic infectious outpatient is diagnosed as COVID-19 case by the hospital with a delay of lag2 days. *ϵ*^2^ is the distribution variance, and *ϵ* was manully assumed to be 600 (around 30% of the number of daily new documented cases at the first peak). We also defined (prop _ doc)_t_, the proportion of documented cases out of total new infections, could be calculated as (prop _ doc)_t_ = p(m| i) × p(hosp _ diag| m)_t_.

Given that the diagnostic capability progressed over time, hospital diagnostic rate p(hosp _ diag| m)_t_ was assumed to have three different values during each of the three periods: p_1_(hosp _ diag| m) is the rate for the period prior to January 27 when test kits were limited, p_2_(hosp _ diag| m) is the rate for the period between January 27 and Feburary 11 when test kits were sufficient but diagnostic criteria was biased without incorporating clinical diagnosis [18], and p_3_(hosp _ diag| m) is the rate for the period after February 12 when test kits were sufficient and diagnostic criteria became more sensitive based on both clinical diagnosis and laboratory diagnosis [16]. The values of p_1_(hosp _ diag| m), p_2_(hosp _ diag| m) and p_3_(hosp _ diag| m) were estimated after fitting the model to the number of daily hospital documented cases. Hospital documented cases on the specific days of January 27, February 12, and February 13, the dates of change in testing capacity [16, 18] (Figure S1), are likely to contain retrospectively documented cases due to the transition to new diagnostic criteria or test kits [29]. Therefore, we removed the original values of these three data and re-filled them by using “na.spline” function in R. That is, the smoothed values of these three dates and the original data of other dates were used during the model fitting process.

### Effective reproductive number **R**_**e**_

After obtaining the posterior distributions of model parameters β_t_, σ, q, γ and model status S_t_, the effective reproductive number R_e_ before and after the intervention policy was implemented can be calculated using the next-generation matrix (NGM) approach. Following methods previously described by Diekmann et al. [30], the transmission matrices T and Σ can be calculated. Briefly, each element in T represents the average number of newly infected cases in the exposed compartment (E) per unit time due to transmission via a single infected individual in the exposed (E) or infectious group (I), calculated as ${\upbeta}_{\mathrm{t}}\left[\left(\frac{\frac{1}{\upsigma}-\upeta}{\frac{1}{\upsigma}}\right)\right]{\mathrm{S}}_{\mathrm{t}}$ or β_t_S_t_. Σ represents the transitions between model states. R_e_ could be calculated as the first eigenvector of the matrix NGM_t_:

5 $$ {\mathrm{N}\mathrm{GM}}_{\mathrm{t}}=\left(\left(-1\right)\left[\begin{array}{cc}\frac{\upbeta_{\mathrm{t}}\left[\left(\frac{\frac{1}{\upsigma}-\upeta}{\frac{1}{\upsigma}}\right)\right]{\mathrm{S}}_{\mathrm{t}}}{\mathrm{N}}& \frac{\upbeta_{\mathrm{t}}{\mathrm{S}}_{\mathrm{t}}}{\mathrm{N}}\\ {}0& 0\end{array}\right]{\left[\begin{array}{cc}-\upsigma & 0\\ {}\upsigma & -\left(\upgamma +\mathrm{q}\right)\end{array}\right]}^{-1}\right) $$

where β_t_, S_t_, σ, q, γ, and N are defined as described above.

R_e_ without the effect of quarantine was calculated as follows: first, we estimated the values of all parameters with quarantine measures through the model fitting process. Second, we simulated the epidemiological curves by setting the quarantine rate as zero (q =0, a scenario without quarantine measures) but keep viral infection-related parameters (σ, η, γ, β_t_) the same as those were estimated. Finally, we calculated this R_e_ through the Eq. (5) using the simulated epidemiological curve (S_t_) and the corresponding parameters above mentioned.

### Model-filters and validations

Since the time-varied true number of individuals in S, E, I, Q and R statuses were not directly observable, we used Particle Markov-chain Monte Carlo (PMCMC) method to handle such hidden variables by simultaneously estimating both the parameters and the hidden variables [31]. Our framework of PMCMC contains two parts: the SEIQR transmission model that generates the transmission dynamics and the observation model that maps SEIQR model to observed hospital document cases. All posterior distributions for the epidemiological hidden variables and parameters were obtained using the PMCMC method, implemented in the Nimble R library [32].

The priors for the parameters were drawn from the following distributions: for the incubation period, 1/σ~U(1, 10); for the latent period, η~U(1, 7); 1/q~U(1, 10), for the time between symptom onset and quarantine start; β_0_~U(0, 1) for the basic transmission rate; and α~N(0, 1), for transportation control coefficient. In the observation model, the priors for time progressed hospital diagnostic rates were set as uniform distribution: p_1_(hosp _ diag| m) /p_2_(hosp _ diag| m) ~U(0, 1), p_2_(hosp _ diag| m) /p_3_(hosp _ diag| m) ~U(0, 1), p_3_(hosp _ diag| m)~U(0, 1).

To assess convergence, three independent chains of the SMC algorithm sets were conducted using 100,000 iterations of 1000 particle samples in each chain. We calculated the effective sample size (ESS) and Gelman-Rubin convergence diagnostic statistics across the three chains.

## Results

### Reconstructing disease dynamics

**Fig. 3:**
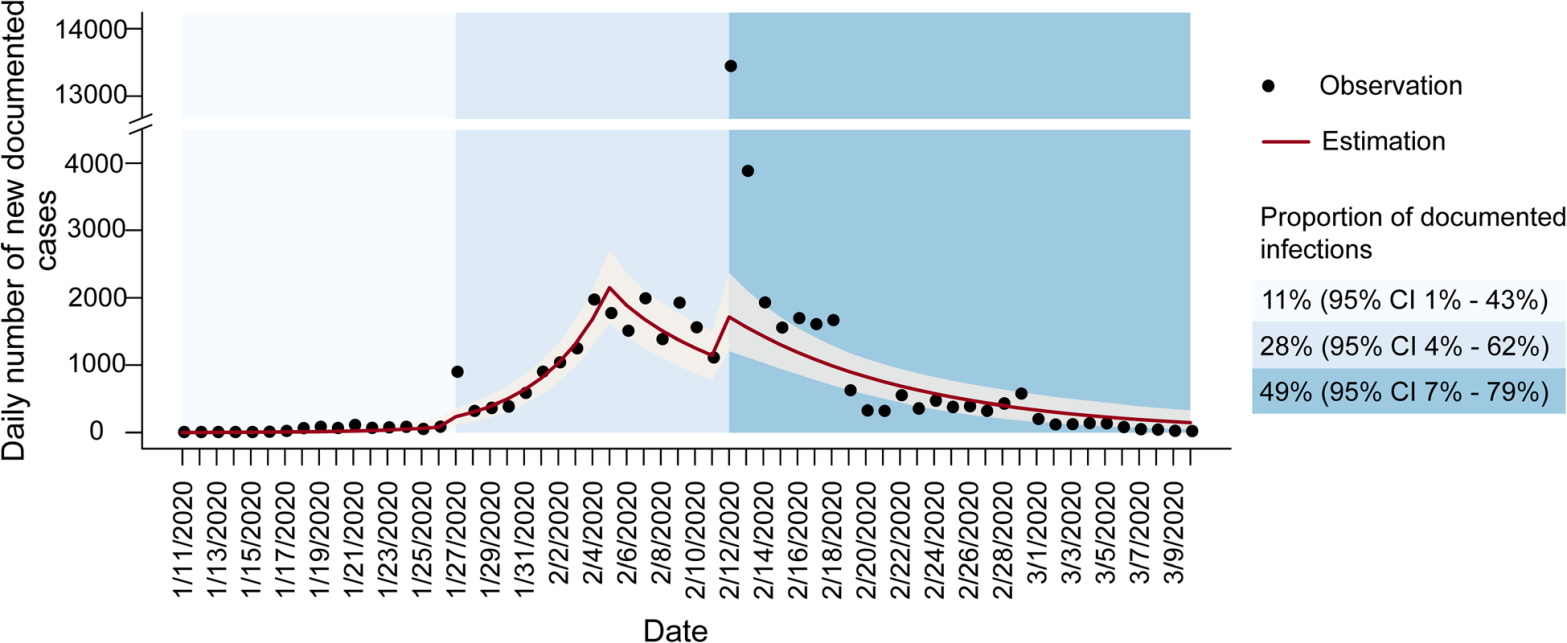
The daily number of new documented confirmed cases by date in Wuhan, China. The red line represents model-estimated cases, grey shadow represents the 95% prediction interval, black points represent the observed documented cases, the blue shaded background denotes incrementally increasing proportions of new documented infections out of total new infections in the corresponding period. Daily documented cases on January 27, February 12, and February 13, the dates of change in testing capacity, are likely to include retrospectively documented cases due to the transition to new diagnostic criteria or test kits [16, 18, 29]. The data on these 3 days were ignored during the model fitting process. A break was made in the y-axis, and the white horizontal bar indicates where the break was set

The daily number of documented COVID-19 cases in Wuhan, increased exponentially up until the first epidemic peak occurring on February 4, and started to fluctuate around the first peak value for about 2 weeks. Note that the values of the highest peak occurring around the end of the second week in two consecutive days in February were ignored in our study because this peak was primarily caused by the retrospectively documented cases under the new diagnostic criteria, whose actual symptom onset date was diversely distributed and can not be traced by our model (Figure S1). The irregular fluctuations can be explained by the effects of interventions and the improved diagnostic capability: the interventions determined the timing of the first peak and may cause a decline pattern afterward; the improved diagnostic capability led to an increase in the number of the documented cases. Together, a high number of cases can be produced for about 2 weeks. Our stochastic SEIQR model reproduced this irregular pattern by a two-peak dynamic with the first peak occurring on February 4 and the second peak occurring shortly on February 12 (Fig. 3). Our estimated times and intensities coincide with the observed epidemic pattern. The estimated incubation period was 5.68 days (95% CI 2.46–8.03), and the estimated latent time was 2.82 days (95% CI 1.10–5.40) (Table 1).

**Table 1:** Parameter estimates of the SEIQR epidemic model. The definitions of the parameters are described. The mean value and 95% credible interval (CI) of the posterior distribution of each of the parameters are included. Convergence is diagnosed to have occurred when the value of Gelman-Rubin convergence is close to 1 or the ESS is larger than 200

Parameters	Definition	Mean	95% CI	Gelman-Rubin convergence	ESS
1/σ	Incubation period (days)	5.68	(2.46, 8.03)	1.006	261.56
η	Latent period (days)	2.82	(1.10, 5.40)	1.005	309.46
1/q	Time between symptom onset and quarantine start (days)	5.44	(1.99, 9.76)	1.003	477.50
α	Transportation restriction coefficient	−1.96	(−2.90, −1.21)	1.003	411.77
β_0_	Basic transmission rate without transportation restrictions	0.67	(0.44, 0.97)	1.001	293.01
p_1_(hosp _ diag| m)	Hospital diagnostic rate from Jan 11 to Jan 26	0.14	(0.01, 0.54)	1.002	396.84
p_2_(hosp _ diag| m)	Hospital diagnostic rate from Jan 27 to Feb 11	0.35	(0.05, 0.78)	1.008	571.52
p_3_(hosp _ diag| m)	Hospital diagnostic rate from Feb 12 to Mar 10	0.61	(0.09, 0.98)	1.004	557.22

### Effects of intervention measures

**Fig. 4:**
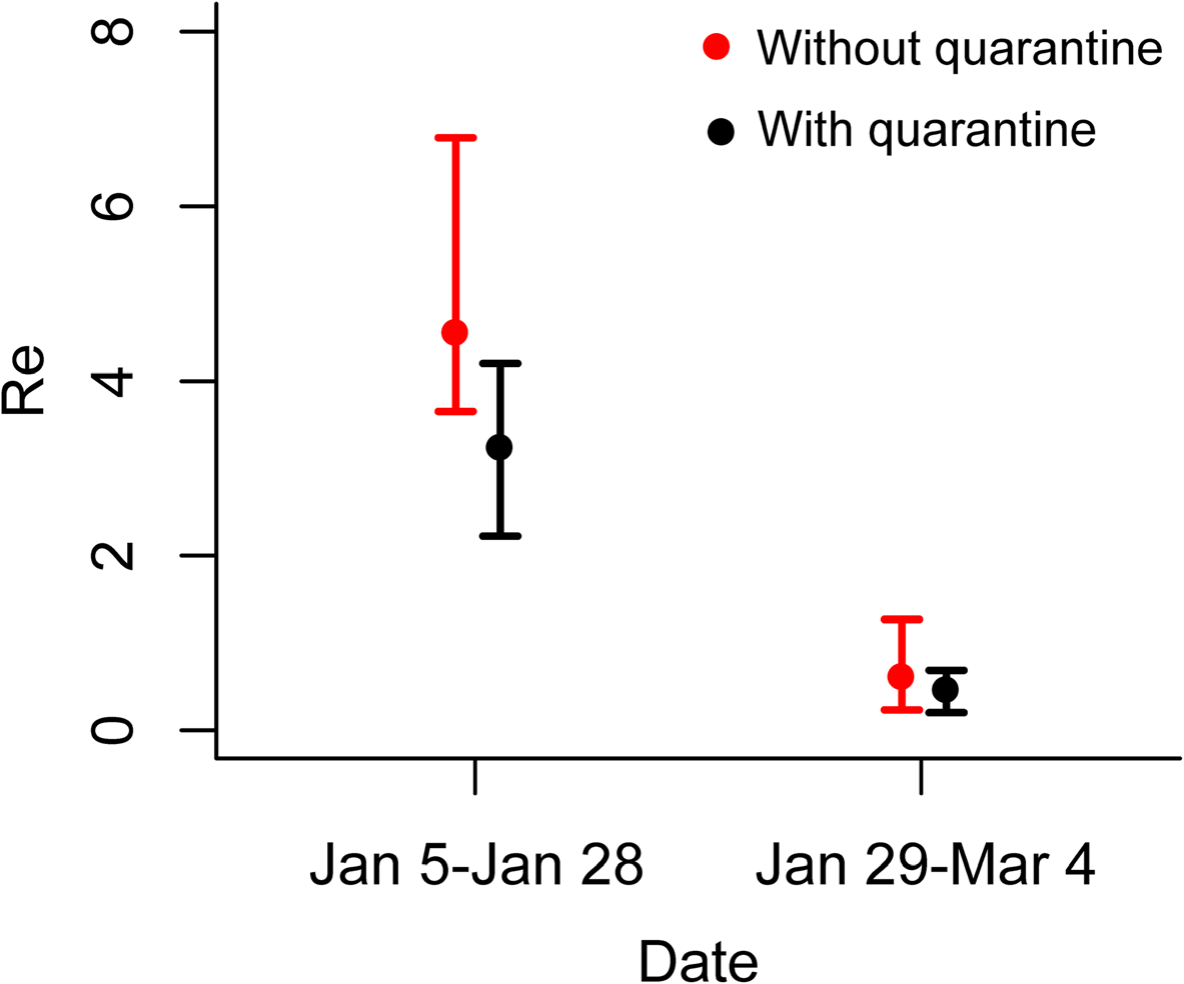
Estimation of the effective reproductive number R_e_ in Wuhan. The red point represents the estimated R_e_ assuming quarantine measures were not implemented, the black point represents R_e_ when quarantine measures were assumed to be implemented, and whiskers show the 95% credible intervals

Both transportation restrictions and quarantine measures had significant impacts on the effective reproductive number R_e_. The initial value of R_e_ was estimated to be 3.23 (95% CI 2.22–4.20) from January 5 to January 28 (Fig. 4), but dropped by 86% to 0.45 (95% CI 0.20–0.69) from January 29 to March 4 after the implementation of transportation restrictions, calculated based on the estimated values of transmission rate β_t_ (Figure S2). The estimated time delay to the start of quarantine after symptom onset was 5.44 days (95% CI 1.99–9.76) (Table 1). For limiting the outbreak growth, quarantine measures were important but not essential. Without quarantine measures, the initial value of R_e_ would increase to 4.54 (95% CI 3.65–6.79) before transportation restrictions had an impact, and would become 0.60 (95% CI 0.23–1.27) after then (Fig. 4). Although R_e_ eventually became less than one, the high initial value of R_e_ would have caused a huge case burden during the early phase of the outbreak. We further tested how the improvements in the diagnostic capacity influenced the estimation of R_e_: about 12–16% overestimation of R_e_ was found due to assuming a fixed diagnostic capacity (Figure S3); and the model fitting Watanabe-Akaike Information Criterion (WAIC) was increased to be 899.50, comparing to 896.06 from our model, indicating a better fit for our model taking account of improving diagnostic capability.

### Effects of detection capability

**Fig. 5:**
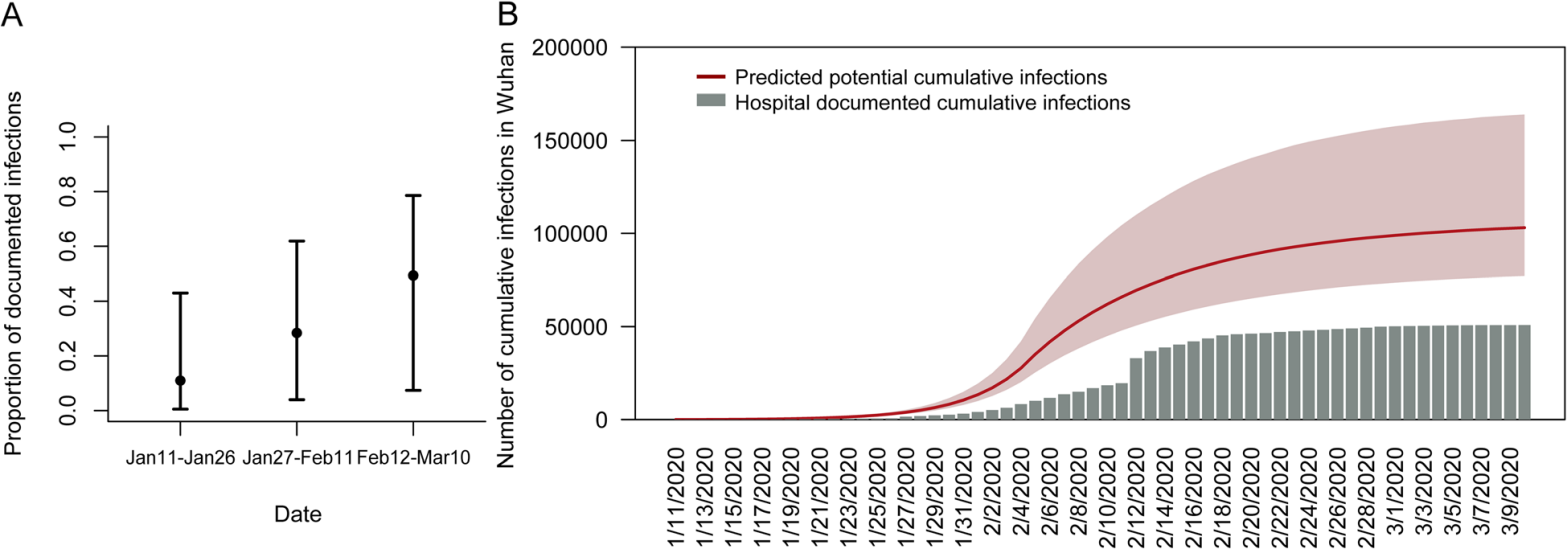
Prediction of temporal diagnostic capability and potential cumulative infections in Wuhan. **a** The estimated proportion of new documented infections out of total new onset infections during different time periods with 95% credible intervals. **b** The red line is the predicted potential total cumulative cases, and the red shadow area represents the 95% prediction interval; the grey bar shows the hospital documented cumulative cases

During the epidemic, the detection capability of COVID-19 in Wuhan was improved several times through the increased availability of test kits and the introduction of more sensitive diagnostic criteria (Fig. 1). These improvements in the detection capability greatly affected the proportion of documented infections during three periods. From January 11 to January 26, the estimated proportion of documented new infections out of total new infections was 11% (95% CI 1–43%), increasing to 28% (95% CI 4–62%) following the increase in test kit production on January 26. Then the proportion increased further to 49% (95% CI 7–79%) after February 12 when more sensitive diagnostic criteria were introduced (Fig. 5a). The estimated potential cumulative number of infections is correlated with but higher than the observed hospital documented cases in Wuhan, and a sudden surge of hospital documented cases on February 12 can be explained by the introduction of the more sensitive diagnostic criteria (Fig. 5b).

## Discussion

This is the first study to demonstrate the effects of intervention measures on the transmission dynamics in Wuhan while taking account of improvements in diagnostic capacity over time. Our results indicated that the transportation restrictions and quarantine measures together in Wuhan were able to contain local epidemic growth by substantially reducing R_e_ by 86%. This proportion of the reduction in R_e_ was exactly the same as the proportion of the reduction in the average daily number of contacts per person (14.6 vs. 2.0) between a baseline period (before the outbreak) and the outbreak period in another study using contact surveys in Wuhan [33]. Since very few studies have estimated the effects of the transportation restrictions in Wuhan, the reduction of contact rate offers valuable information to project the possible effects on the reproduction number. Assuming the transmissibility was proportional to the contact numbers, the reduction ratio of the contact numbers will be proportional to the reduction ratio in R_e_. These results confirm that measuring contact mixing is an accurate way to estimate the impacts of intervention measures. Furthermore, the proportion of undocumented infections was estimated to be reduced during the outbreak, as a consequence of the improvements in diagnostic capability. These findings will help to inform further analysis aimed at developing prevention strategies and evaluating the effects of public health interventions.

While most studies assumed a fixed proportion of documented infections over time, the study presented here estimates an initial proportion of documented infections of 11%, similar to previous predictions of 14% by Ruiyun et al. [17], which progressively increases with the improvement of diagnostic capability. Our results suggest that the increase in the number of cases during the early outbreak needs to be interpreted cautiously, given that the proportion of documented infections is highly dependent on the availability and use of test kits over time. As detection was enhanced through improved clinical diagnosis [16], a sharp rise in cumulative cases on February 12 is likely explained by prior onset cases retrospectively documented under new diagnostic criteria. The undocumented infections may be largely associated with mild illness that are insufficiently serious to seek treatment [17]. Our results show that the estimated proportion of documented new infections out of total new infections increased to 49% after diagnostic sensitivity was increased. Besides the increased test kit production and the more sensitive diagnostic criteria mentioned above, there are other factors that may enhance case detection: such as extensive testing, more test equipment, and more health workers and expertise [34]. Over the study period, the amount of community testing was strongly dependent on the supply of test kits [35], especially for the time before January 26. The amount of test equipment/health workers and expertise was gradually increased over time, however, the related data is not available.

The estimation of R_e_ in the study from January 5 to January 28 is consistent with other recent studies [36] (3.11 by Jonathan et al. [5], 3.15 by Tian et al. [6], 1.4 to 3.9 by Li et al. [1], see in Table 2). Furthermore, our results demonstrate that the combination of transportation restrictions and quarantine measures was able to reduce COVID-19 transmission. Transportation restrictions, including stopping all forms of public transportation, including trains, and air travel, sharply reduced social contacts thereby reducing virus transmission rates [13, 17]. Population behavioral responses (e.g., social distancing, contacts mixing, wearing facemasks, etc.) were changed concurrently with the implementation of transportation measures [33, 38]. Because a gradual increase in documented hospital cases in February can be partly due to the increased detection capability, the effect of intervention measures (indicated as the reduction in R_e_) was estimated to be larger than previous studies that assumed fixed detection rates over the course of the epidemic. For example, R_e_ was estimated to drop by 55.3% by Kucharski et al. [13]. Quarantine of infections was also found to be essential in curbing the epidemic. Our model estimated that the time between symptom onset and quarantine start was 5.44 days, similar to the estimates previously reported by Tian et al. (5.19 days) [6].

**Table 2:** A summary of models, data descriptions, reported estimates of the basic/effective reproductive number

Ref.	Model	Data (study period)	Basic (R_0_) or effective (R_e_) reproduction number
Li et al. [1]	stochastic standard susceptible-exposed infectious-recovered (SEIR) model	daily onset cases in Wuhan, China (December 10–January 4, 2020)	2.2 (95% CI: 1.4–3.9)
Jonathan et al. [5]	deterministic susceptible-exposed infectious-recovered (SEIR) metapopulation model	daily reported cases in Wuhan, China (January 1–January 22, 2020)	3.11 (95% CI: 2.39–4.13)
Tian et al. [6]	deterministic susceptible-exposed infectious-recovered (SEIR) model	daily reported cases in 262 cities in China, including Wuhan (December 31, 2019 - February 19, 2020)	3.15 (95% CI: 3.04–3.26, before the implementation of the transportation restrictions); 0.97–3.05 (after control was scaled-up from 23 January onward)
Majumder et al. [37]	incidence decay and exponential adjustment (IDEA) model	daily reported cases in Wuhan, China (December 1, 2019 - January 26, 2020)	2.54–3.61
Kucharski et al. [13]	meta-population stochastic susceptible-exposed infectious-recovered (SEIR) model	daily onset cases in Wuhan and internationally exported cases from Wuhan, China (December 1, 2019 - February 10, 2020)	2.35 (95% CI: 1.15–4.77, 1 week before transportation restrictions were introduced); 1.05 (95% CI: 0.41–2.39, 1 week after transportation restrictions were introduced)

The estimated incubation period was 5.68 days which is also consistent with other recent studies [1, 6, 39–41]. As the estimated latent period is 2.82 days, some transmissions may occur before the symptom onset. Finding ways to reduce possible contact during the pre-symptomatic transmission period may be a critical component in containing the spread of the virus. Given the existence of pre-symptomatic transmission, this study supports government recommendations that people who have had close contact with confirmed cases, regardless of whether they show symptoms or not, need to be quarantined for 14 days [42].

The current study suggests that although intensive transportation restrictions and quarantine measures were critical in containing the COVID-19 outbreak in Wuhan, the improvements in detection capability have to be taken into account in order to evaluate the effectiveness of these intervention measures more accurately. This will allow more meaningful evaluations of public health control effects, which are important for making decisions on which intervention used in Wuhan should be replicated in other parts of the world in order to effectively control the current pandemic.

There are two limitations to this study. First, in addition to its effect on the infected individuals, the quarantine intervention can result in a lower number of susceptible individuals. Our model did not consider that because the number of close contacts during that period is not available. Given that the daily incidence was about 200 cases per million population and that Wuhan’s population size is approximatly 11 million [26], the proportion of susceptible individuals that were traced and quarantined each day is relatively small if we assume each infected case contacted 30 individuals. Its impact on the estimation of R_e_ would be therefore small. Second, in this study, we assumed that the proportion of asymptomatic cases among all cases was constant over time. It is unlikely that it will be possible to get good estimates of the number of asymptomatic cases during the outbreak. Despite these limitations, we demonstrated that our methodology allowed for improved approximation of the actual epidemic pattern by taking account of changes in diagnostic capacity.

## Conclusions

The combination of transportation restrictions and quarantine measures used in Wuhan was able to effectively contain local COVID-19 epidemic spread.

## Supplementary Information

**Supplementary Figures: Figure S1.** The original observed daily documented cases without removing values. The red points indicate the observed number of cases at the dates when many retrospectively documented cases were counted. Data in these 3 days were replaced by smoothing values because they contain many retrospectively documented cases. The black points indicate the observed number of cases. Blue shaded background denotes incrementally increasing proportions of new documented infections out of total new infections on the corresponding period caused by improved diagnostic rates. **Figure S2.** Estimation of the transmission rate β_t_ with 95% credible intervals. **Figure S3.** Estimation of the effective reproductive number R_e_ using a fixed hospital diagnostic rate in Wuhan. The fixed hospital diagnostic rate was assumed to be equal to the estimated mean value of the original rate (0.14, see in Table 1) when not considering the improvement of diagnostic capability. R_e_ was estimated to be 3.76 (95% CI 2.43 - 4.36) before the transportation restrictions worked and to be 0.56 (95% CI 0.34 - 0.79) after then. **Figure S4.** Trace plots of parameter values for the model frame. The three different colours represent three chains.

## Data Availability

The data and materials are available from the corresponding author on reasonable request.

## Abbreviations

SEIQR: Susceptible-exposed-infected-quarantined-recovered
PMCMC: Particle markov-chain monte carlo
SFDA: The state food and drug administration
NGM: Next-generation matrix
WAIC: Watanabe-akaike information criterion
CI: Credible interval
IDEA: Incidence decay and exponential adjustment model
