# Supplementary material for: Estimating effects of intervention measures on COVID-19 outbreak in Wuhan taking account of improving diagnostic capabilities using a modelling approach
Source: BMC Infect Dis. 2021 May 5;21:424. doi: 10.1186/s12879-021-06115-6 (PMC8097251; doi:10.1186/s12879-021-06115-6)
Supplement: Supplementary file 1 — Supplementary Figures: Figure S1. The original observed daily documented cases without removing values. The red points indicate the observed number of cases at the dates when many retrospectively documented cases were counted. Data in these 3 days were replaced by smoothing values because they contain many retrospectively documented cases. The black points indicate the observed number of cases. Blue shaded background denotes incrementally increasing proportions of new documented infections out of total new infections on the corresponding period caused by improved diagnostic rates. Figure S2. Estimation of the transmission rate βt with 95% credible intervals. Figure S3. Estimation of the effective reproductive number Re using a fixed hospital diagnostic rate in Wuhan. The fixed hospital diagnostic rate was assumed to be equal to the estimated mean value of the original rate (0.14, see in Table 1) when not considering the improvement of diagnostic capability. Re was estimated to be 3.76 (95% CI 2.43 - 4.36) before the transportation restrictions worked and to be 0.56 (95% CI 0.34 - 0.79) after then. Figure S4. Trace plots of parameter values for the model frame. The three different colours represent three chains. [file 12879_2021_6115_MOESM1_ESM.docx]

**Supplementary Material**

**Estimating effects of intervention measures on COVID-19 outbreak in Wuhan taking account of improving diagnostic capabilities using a modelling approach**

Jingbo Liang^1^, Hsiang-Yu Yuan^1*^, Lindsey Wu^2^, Dirk  Udo Pfeiffer^3^

^1^Department of Biomedical Sciences, Jockey Club College of Veterinary Medicine and Life Sciences, City University of Hong Kong, Hong Kong

^2^Department of Infection Biology, Faculty of Infectious and Tropical Diseases, London School of Hygiene & Tropical Medicine, United Kingdom

^3^Centre for Applied One Health Research and Policy Advice, City University of Hong Kong, Hong Kong, China

^*^Correspondence to: Hsiang-Yu Yuan [sean.yuan@cityu.edu.hk](mailto:sean.yuan@cityu.edu.hk)

**

**

Figure S1. The original observed daily documented cases without removing values. The red points indicate the observed number of cases at the dates when many retrospectively documented cases were counted. Data in these three days were replaced by smoothing values because they contain many retrospectively documented cases. The black points indicate the observed number of cases. Blue shaded background denotes incrementally increasing proportions of new documented infections out of total new infections on the corresponding period caused by improved diagnostic rates.


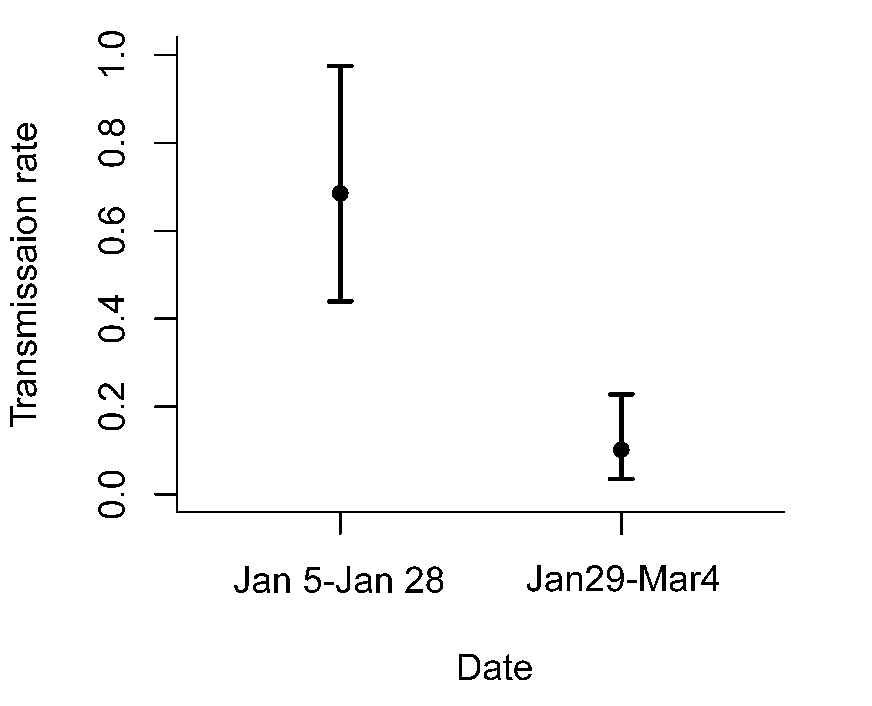


Figure S2. Estimation of the transmission rate $\beta_{t}$ with 95% credible intervals.


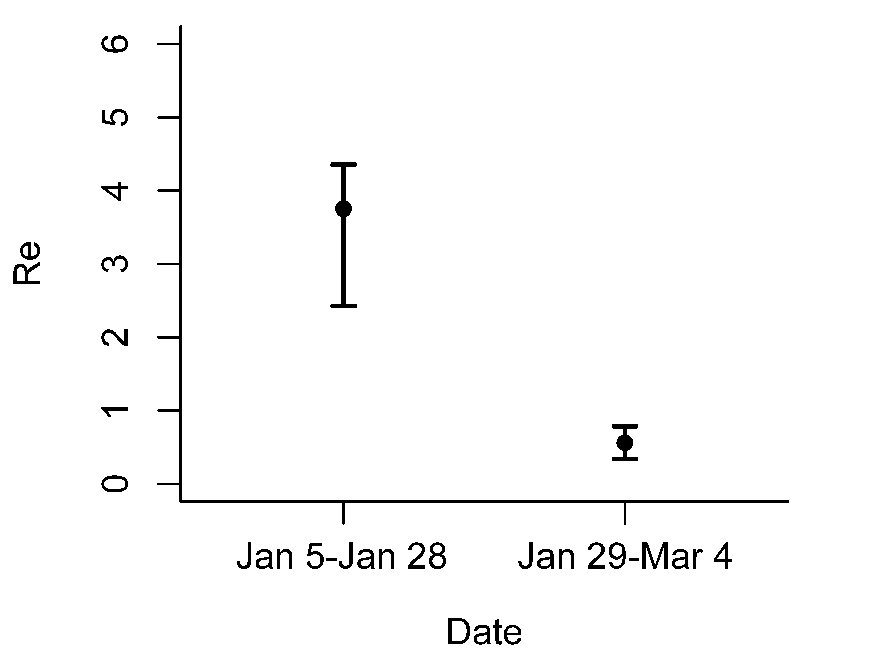


Figure S3. Estimation of the effective reproductive number $R_{e}$ using a fixed hospital diagnostic rate in Wuhan. The fixed hospital diagnostic rate was assumed to be equal to the estimated mean value of the original rate (0.14, see in Table 1) when not considering the improvement of diagnostic capability. $R_{e}$ was estimated to be 3.76 (95% CI 2.43 - 4.36) before the transportation restrictions worked and to be 0.56 (95% CI 0.34 - 0.79) after then.
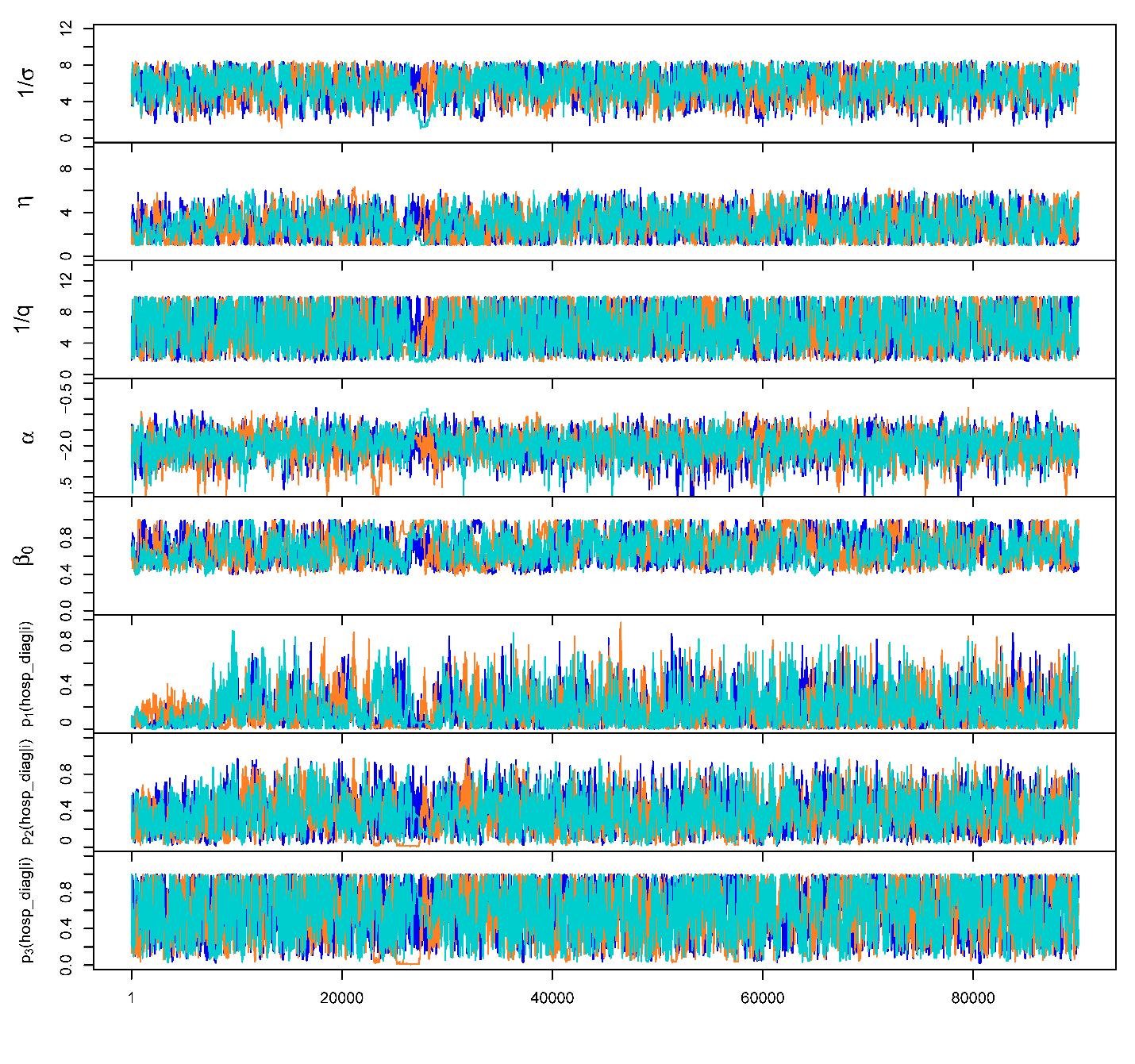


Figure S4. Trace plots of parameter values for the model frame. The three different colours represent three chains.
